# Relationship between Cancer Worry and Stages of Adoption for Breast Cancer Screening among Korean Women

**DOI:** 10.1371/journal.pone.0132351

**Published:** 2015-07-17

**Authors:** Eunji Choi, Yoon Young Lee, Hyo Joong Yoon, Sangeun Lee, Mina Suh, Boyoung Park, Jae Kwan Jun, Yeol Kim, Kui Son Choi

**Affiliations:** 1 Graduate School of Cancer Science and Policy, National Cancer Center, Goyang, Gyeonggi-do, Republic of Korea; 2 National Cancer Control Institute, National Cancer Center, Goyang, Gyeonggi-do, Korea; Iranian Institute for Health Sciences Research, ACECR, ISLAMIC REPUBLIC OF IRAN

## Abstract

**Background:**

The possibility of developing breast cancer is a concern for all women; however, few studies have examined the relationship between cancer worry and the stages of adoption for breast cancer screening in Korea. Here, we investigated the associations between cancer worry, the stages of adopting breast cancer screening, and socio-demographic factors known to influence screening behaviors.

**Methods:**

This study was based on the 2013 Korean National Cancer Screening Survey, an annual cross-sectional survey that utilized nationally representative random sampling to investigate cancer screening rates. Data were analyzed from 1,773 randomly selected women aged 40–74 years. Chi-squared tests and multinomial logistic analyses were conducted to determine the associations between cancer worry and the stages of adoption for breast cancer screening and to outline the factors associated with each stage.

**Results:**

Korean women were classified into the following stages of adoption for breast cancer screening: pre-contemplation (24.7%), contemplation (13.0%), action/maintenance (50.8%), relapse risk (8.9%), and relapse (2.6%). Women in the action/maintenance stages reported more moderate to higher levels of worry about getting cancer than those in the pre-contemplation stage. Further, age of 40–49 years and having private cancer insurance were associated with women in the action/maintenance stages.

**Conclusion:**

Interventions to address breast cancer worry may play an important role in increasing participation and equity in breast cancer screening.

## Introduction

Breast cancer is one of the most common cancers around the world. According to GLOBOCAN fact sheets, 25% of all of the new cancer cases involve the breast. While breast cancer is the most frequent cancer among women of both highly and less developed nations [1], there are considerable differences in prognoses for breast cancer between individual countries: in highly developed countries, breast cancer survival (between 85% and 90%) tends to be much higher than that for less developed countries [2]. Much of the actual variations in breast cancer survival between countries are attributable to the availability of screening programs and access to appropriate treatment services. In particular, breast cancer screening using mammography is widely considered to be instrumental to improving survival rates from breast cancer.

In Korea, even though the incidence of breast cancer is not as high as that of Western countries, it is rapidly increasing. Breast cancer is the second most common type of cancer and the fifth leading cause of cancer death among Korean women [3]. In Korea, the National Health Insurance (NHI), compulsory social insurance, covers all residents living within the country. Additionally, the Medical Aid (MA) program provides medical services for low-income households [4]. In 2002, Korea began nationwide breast cancer screening as a part of the National Cancer Screening Program (NCSP) to provide biennial mammographic screening for women aged 40 years and over [5]. Via the NCSP, MA enrollees and people covered by the NHI with a contribution below 50% are eligible for breast cancer screening free-of-charge, while the remaining NHI beneficiaries are eligible for breast cancer screening with a co-payment of 10% of the cost of the mammographic test.

Although organized breast cancer screening is offered, breast cancer screening rates for Korea (70.9% for the entire nation) are still below those for other developed countries [6, 7]. In a previous study, “fear of cancer detection” was one of several reasons given by Korean women for noncompliance with breast cancer screening [6]. While many studies have identified predictors of adherence to breast cancer screening guidelines, little is known about the association between cancer worry and breast cancer screening uptake. In the literature, cancer worry is defined as an emotional reaction to the threat of cancer [1,8, 9], and four theoretical hypotheses related to the effects of cancer worry on screening behavior have been proposed: (1) cancer worry facilitates screening [10–12], (2) cancer worry inhibits screening [13], (3) a moderate level of cancer worry, neither too high nor too low, optimizes screening [14], and (4) cancer worry encourages screening only in the presence of moderating factors, such as self-efficacy [9]. Although some studies have reported contradictory results, many suggest that higher levels of cancer worry are associated with increased odds of undergoing cancer screening. Thus, better understanding of the influence of concerns for getting cancer on screening behaviors may help with developing patient-focused interventions with which to efficiently increase cancer screening rates.

In addition to achieving high initial rates, the maintenance of participation rates at subsequent rounds of screening is equally as important [15]. Accordingly, many studies have applied transtheoretical models to evaluate changes in attitudes related to continued cancer screening [16–20]. Transtheoretical models provide a methodological approach with which to investigate awareness and willingness to adopt health behaviors in a given population [19, 21]. The results thereof are reported as stages of adopting the targeted behavior (e.g., undergoing continued cancer screening).

In this study, via a transtheoretical model, we aimed to examine the stages of adoption for breast cancer screening to outline the extents to which breast cancer worry affect the decisions of Korean women to undergo breast cancer screening or not. We also investigated associations between the stages of adopting breast cancer screening and socio-demographic factors known to influence screening behaviors. We suspect that providing detailed information on stages of adoption and factors influencing breast cancer screening will help researchers tailor health communication to increase participation in breast cancer screening.

## Materials and Methods

### Study population

Data were acquired from subjects included in the 2013 Korean National Cancer Screening Survey (KNCSS). The KNCSS is an annual cross-sectional survey performed to investigate screening rates among Koreans for five common cancers (gastric, liver, colorectal, breast, and cervix) through nationally representative random sampling. Men aged ≥40 years and women ≥30 years were selected based on Resident of Registration Population data for July 2013 compiled by Statistics Korea, using multistage random sampling according to sex, age, geographic area, and size of population per area. The survey was conducted from September 26 to October 18, 2013, at which time investigators from a professional research agency went door-to-door to recruit residents. At least three attempts were made to contact individual residents. The 2013 KNCSS included 4,100 participants among a total of 23,238 random samples (total response rate: 17.6%). The response rate after making contact was 64%, excluding those who were absent (16,533) or who did not meet the criteria for this survey (296). Of the respondents, 1,773 cancer-free women aged ≥40 years were finally included in the current study, since the NCSP only provides breast cancer screening for individuals aged ≥40 years.

### Ethics Statement

Witten informed consent was obtained from all study participants before the face-to-face interview. This study was approved by the Institutional Review Board (IRB) of the National Cancer Center, Korea (approval number: NCCNCS-08-129). Informed consent was documented via the use of a written consent form approved by the IRB and signed by all study participants.

### Measurement

The stages of adoption for breast cancer screening were assessed via a modified five-stage process related to the subjects’ reported histories of breast cancer screening and their current intentions to undergo screening within the next 2 years, as recommended by Rakowski et al. in 1996 [20]. The participants were classified as follows:

1. Pre-contemplation, had never undergone breast cancer screening and did not plan to within the next 2 years;
2. Contemplation, had either never undergone breast cancer screening or had not in the last 1–2 years, but are planning to within the next 2 years;
3. Action/Maintenance, had undergone breast cancer screening within the last 24 months and were planning on undergoing another test within the next 2 years;
4. Relapse risk, had undergone breast cancer screening within the last 24 months, but were not planning on undergoing another test within the next 2 years;
5. Relapse, had undergone one or more breast cancer screening test in the past, but were now off schedule and had no plans to undergo breast cancer screening within the next 2 years.

To develop survey questions related to cancer worry and perceived risk, we reviewed previous research. Since measurements of cancer worry and perceived risk were not available in the Korean language, questionnaires written in English were first translated into Korean and then translated back into English to confirm the accuracy of the translation. Further, we conducted a pilot survey to assess understanding of these questions among respondents. In the present study, Lerman’s Cancer Worry Scale modified to reflect cancer worry in Korea was used [22, 23, 24, 25]. Overall cancer worry was assessed via two questions: (1) “How often have you worried about your own chances of developing cancer?” and (2) “How often have you thought about your own chances of developing cancer?” The worry items used in this study refer to frequency (i.e., “How often do you worry?”) rather than magnitude (i.e., “How much do you worry?”). For each question, participants were asked to respond via the five following scaled response items: (1) never, (2) rarely, (3) sometimes, (4) often, and (5) always. Cancer worry was calculated as the total sum of the scores for both questions divided by two, with possible scores ranging from 1 to 5. Cancer worry levels were then divided into three groups: low (score of 1–2), middle (score of 3), and high (score of 4–5). Further, we assessed worry levels for five major non-communicable diseases: Alzheimer’s, stroke, hypertension, cardiovascular disease, and diabetes. As a slightly different concept from cancer worry, “perceived risk for breast cancer” was also evaluated to determine its influence on cancer worry and stages of adoption via the following question: “Compared with the average person of your age, what do you think your chances are of developing breast cancer?” The question was derived from an article on developing instruments to measure psychological factors affecting colorectal cancer screening [25, 26]. Participants were asked to report their perceived risk on a five-point scale, with higher scores reflecting greater perceived risk. The levels of perceived risk were also divided into three groups: low (score of 1–2), middle (score of 3), and high (score of 4–5).

We also examined several socioeconomic (age, marital status, level of education, and monthly household income) and health-related factors (regular exercise, undergoing regular health checkups, and family history of any cancer) to assess their influence on screening behaviors. Further, private cancer insurance and awareness of the NCSP were also measured to assess the accessibility of breast cancer screening. Although the NHIS provides universal coverage to all Koreans, many Koreans pay for supplementary private health insurance in the event of illness (e.g., cancer or stroke) due to insufficient coverage with the NHIS [4].

### Statistical analysis

Descriptive statistics were assessed to characterize the study population. For comparison of cancer worry levels with worry levels for five major non-communicable diseases, ANOVA and Tukey’s post-hoc analysis were conducted. Chi-squared tests were used to compare the distributions of the stages of adoption according to socioeconomic and health-related factors. To investigate factors related to one’s stage of adoption, we grouped participants into four groups: 1) pre-contemplation; 2) contemplation; 3) action/maintenance; and 4) relapse risk/relapse group. Multinomial logistic analysis was conducted to identify associations between breast cancer worry and the stages of adoption for breast cancer screening, as well as factors associated with each stage. In this analysis, we combined the relapse risk and relapse stages, because the number of women who were in the relapse stage was too small to conduct multivariate analysis. Further, women in the relapse risk and relapse share the same experience in that they have already been in action or maintenance. We assessed the adjusted odds of being in the action/maintenance group or relapse risk/relapse group rather than the pre-contemplation group. All variables with a *P*-value of <0.05 in univariate analysis were included in the multinomial logistic regression model as potential predictors. These variables included age, monthly household income, residence area, private cancer insurance, regular health checkups, and cancer worry. Additionally, we conducted trend analysis for the ordinal categorical variables to investigate the significance of any trends. Statistical analyses were performed using STATA software (version 13; StataCorp. L.P., College Station, TX), and all *P*-values <0.05 were considered statistically significant.

## Results

Descriptive data for the subjects according to their levels of cancer worry are presented in Table 1. The mean age of the 1,773 respondents was 53.7 years. Nearly 50% of the participants were included in the middle household income group, below $4990 per month; about 23% of the participants had acquired an advanced college degree; and most of the participants were married. Regarding cancer worry, significant differences were noted in the distributions of cancer worry levels according to age group; elderly women showed the highest levels of cancer worry. Additionally, cancer worry levels differed according to awareness of the NCSP. Compared to those who did not know of its existence, those who did comprised a significantly greater proportion of individuals highly worried about getting cancer.

**Table 1:** Sociodemographic characteristics of the study population (n = 1,773; Korea, 2013) according to cancer worry.

		Cancer Worry			
	n (%)	High	Moderate	Low	*P*-value
Total	1773(100.0)	477(26.9)	622(35.1)	674(38.0)	
Age(years)					0.006
40–49	665(37.5)	159(23.9)	262(39.4)	244(36.6)	
50–59	605(34.1)	165(27.2)	188(31.0)	252(41.6)	
60–74	503(28.3)	153(30.4)	172(34.2)	178(35.4)	
Monthly Household Income ($)					0.396
<2500	448(25.3)	128(28.5)	140(31.2)	180(40.1)	
2500–4990	763(43.0)	198(25.9)	276(36.1)	289(37.8)	
>5000	561(31.6)	151(26.9)	205(36.5)	205(36.5)	
Education					0.400
Middle and high school graduate	1367(77.1)	363(26.5)	491(35.9)	513(37.5)	
Undergraduate college degree or higher	406(22.9)	114(28.0)	131(32.2)	161(39.6)	
Marital Status					0.661
Other	171(9.6)	50(29.2)	61(35.6)	60(35.0)	
Married	1602(90.4)	427(26.6)	561(35.0)	614(38.3)	
Residential Area					0.351
Urban	791(44.6)	224(28.3)	276(34.8)	291(36.7)	
Suburban	733(41.3)	183(24.9)	269(36.7)	281(38.3)	
Rural	249 (14.0)	70 (28.1)	77 (30.9)	102 (40.9)	
Private Cancer Insurance					0.441
Yes	1453(82.0)	389(26.7)	502(34.5)	562(38.6)	
No	320(18.0)	88(27.5)	120(37.5)	112(35.0)	
Family History of Cancer					0.062
Yes	467(26.3)	144(30.8)	149(31.9)	174(37.2)	
No	1306(73.7)	333(25.5)	473(36.2)	500(38.2)	
Knowledge of the NCSP					0.019
Yes	1395(78.6)	388(27.8)	500(35.8)	507(36.3)	
No	378(21.3)	89(23.5)	122(32.2)	167(44.2)	
Regular Exercise					0.962
Yes	831(46.9)	221(26.5)	293(35.2)	317(38.1)	
No	942(53.1)	256(27.1)	329(34.9)	357(37.9)	
Regular Health Checkups					0.781
Yes	849(47.9)	223(26.2)	304(35.8)	322(37.9)	
No	924(52.1)	254(27.4)	318(34.4)	352(38.1)	
Perceived Risk for Breast Cancer					0.973
High	402(22.7)	106(26.3)	143(35.5)	153(38.0)	
Moderate	974(54.9)	265(27.2)	344(35.3)	365(37.4)	
Low	397(22.4)	106(26.7)	135(34.0)	156(39.2)	

NCSP, National Cancer Screening Program

Table 2 lists mean worry levels among Korean women for six major non-communicable diseases. With statistical significance, the mean level of cancer worry was higher than the mean levels for all of the other non-communicable diseases.

**Table 2:** Worry levels among Korean women for six major non-communicable diseases.

Non-communicable disease	Mean ± s.d.	*P*-value
Cancer	3.05 ± 0.84	<0.00
Alzheimer’s ^a^	2.66 ± 0.99	
Stroke ^a^	2.65 ± 0.99	
Hypertension ^a^	2.63 ± 0.98	
Cardiovascular disease ^a^	2.55 ± 0.91	
Diabetes ^a^	2.49 ± 0.98	

^a^The mean difference between cancer worry and each disease is statistically significant at a level of significance of *P*<0.05, ANOVA with Tukey’s test

In the present study, we classified respondents according to stage of adoption for breast cancer screening. Among the study population, 24.7% were classified in the pre-contemplation stage, 13.0% in the contemplation stage, 50.8% in the action/maintenance stage, 8.9% in the relapse risk stage, and 2.6% in the relapse stage. Age, monthly household income, private cancer insurance, regular health checkups, and cancer worry were significantly different across the stages of adoption of breast cancer screening (Table 3).

**Table 3:** Stage of adoption of breast cancer screening according to sociodemographic characteristics of the population (n = 2364; Korea, 2013).

	Pre-contemplation	Contemplation	Action / Maintenance	Relapse risk	Relapse	*P*-value
	n (%)	n (%)	n (%)	n (%)	n (%)	
Total	438 (24.7)	231 (13.0)	901 (50.8)	157 (8.9)	46 (2.6)	
Age(years)						0.043
40–49	141 (21.2)	85 (12.7)	371 (55.7)	53 (7.9)	15 (2.2)	
50–59	158 (26.1)	74 (12.2)	305 (50.4)	53 (8.7)	15 (2.4)	
60–74	139(27.6)	72(14.3)	225(44.7)	51(10.1)	16(3.2)	
Monthly Household Income ($)						0.032
<2500	111 (24.7)	66 (14.7)	213 (47.5)	40 (8.9)	18 (4.0)	
2500–4990	209 (27.3)	97 (12.7)	373 (48.8)	66 (8.6)	18 (2.3)	
>5000	117 (20.8)	68 (12.1)	315 (56.1)	51 (9.0)	10 (1.7)	
Education						0.565
Middle and high school graduate	346 (25.3)	169 (12.3)	695 (50.8)	121 (8.8)	36 (2.6)	
Undergraduate college degree or higher	92 (22.6)	62 (15.2)	206 (50.7)	36 (8.8)	10 (2.4)	
Marital Status						0.112
Other	45 (26.3)	32 (18.7)	73 (42.6)	16 (9.3)	5 (2.9)	
Married	393 (24.5)	199 (12.4)	828 (51.6)	141 (8.8)	41 (2.5)	
Residential Area						0.028
Urban	182 (23.0)	83 (10.4)	425 (53.7)	82 (10.3)	19 (2.4)	
Suburban	194 (26.4)	109 (14.8)	351 (47.8)	59 (8.0)	20 (2.7)	
Rural	62 (24.9)	39 (15.6)	125 (50.2)	16 (6.4)	7 (2.8)	
Private Cancer Insurance						0.026
Yes	339 (23.3)	198 (13.6)	753 (51.8)	128 (8.8)	35 (2.4)	
No	99 (30.9)	33 (10.3)	148 (46.2)	29 (9.0)	11 (3.4)	
Family History of Cancer						0.311
Yes	125 (24.7)	49 (10.4)	235 (50.3)	45 (9.6)	13 (2.7)	
No	313 (23.9)	182 (13.9)	666 (51.0)	112 (8.5)	33 (2.5)	
Knowledge of the NCSP						0.073
Yes	328(23.5)	189(13.3)	725(51.9)	117(8.4)	39(2.8)	
No	110(29.1)	45(11.9)	176(46.5)	40(10.5)	7(1.8)	
Regular Exercise						0.056
Yes	220 (26.4)	119 (14.3)	395 (47.5)	71 (8.5)	26 (3.1)	
No	218 (23.1)	112 (11.8)	506 (53.7)	86 (9.1)	20 (2.1)	
Regular Health Checkups						0.010
Yes	209 (24.6)	98 (11.5)	462 (54.4)	64 (7.5)	16 (1.8)	
No	229 (24.7)	133 (14.3)	439 (47.5)	93 (10.0)	30 (3.2)	
Cancer Worry						0.008
High	116 (24.3)	47 (9.8)	250 (52.4)	50 (10.4)	14 (2.9)	
Moderate	134 (21.5)	81 (13.0)	344 (55.3)	48 (7.7)	15 (2.4)	
Low	188 (27.8)	103 (15.2)	307 (45.5)	59 (8.7)	17 (2.5)	
Perceived Risk for Breast Cancer						0.662
High	90 (22.3)	55 (13.6)	214 (53.2)	35 (8.7)	8 (1.9)	
Moderate	246 (25.2)	121 (12.4)	500 (51.3)	83 (8.5)	24 (2.4)	
Low	102 (25.6)	55 (13.8)	187 (47.1)	39 (9.8)	14 (3.5)	

NCSP, National Cancer Screening Program

Table 4 lists the factors shown to be associated with the stages of adoption for breast cancer screening. In this analysis, we combined the relapse risk and the relapse stages. Analyzed via multinomial logistic regression using the pre-contemplation as the reference category, women who had moderate to high levels of cancer worry were more likely to be in the action/maintenance stage than the pre-contemplation stage (*P* for trend = 0.021). Meanwhile, no statistically significant difference in cancer worry levels was noted among women in the pre-contemplation, contemplation, and relapse risk/relapse stages.

**Table 4:** Multinomial logistic analysis of factors associated with each stage of adoption for breast cancer screening in Korea, 2013^a^.

	Contemplation			Action/Maintenance			Relapse risk or Relapse		
	aOR	95% CI	*P* for trend	aOR	95% CI	*P* for trend	aOR	95% CI	*P* for trend
Age(years)									
40–49	1.13	0.75–1.68	0.627	1.64	1.22–2.20	0.001	1.01	0.66–1.52	0.841
50–59	0.89	0.59–1.32		1.23	0.92–1.64		0.90	0.59–1.36	
60–74	1.00			1.00			1.00		
Monthly Household Income ($)									
<2500	0.76	0.51–1.13	0.845	0.86	0.64–1.15	0.051	0.73	0.48–1.13	0.859
2500–4990	0.94	0.61–1.45		1.30	0.95–1.79		0.95	0.60–1.49	
>5000	1.00			1.00			1.00		
Residential Area									
Urban	0.72	0.45–1.17	0.150	1.12	0.78–1.59	0.219	1.51	0.88–2.60	0.076
Suburban	0.88	0.55–1.41		0.84	0.59–1.21		1.12	0.64–1.94	
Rural	1.00			1.00			1.00		
Private Cancer Insurance									
Yes	1.73	1.12–2.67		1.43	1.07–1.92		1.19	0.78–1.81	
No	1.00			1.00			1.00		
Regular Health Checkups									
Yes	0.79	0.57–1.10		1.18	0.94–1.49		0.71	0.51–1.01	
No	1.00			1.00			1.00		
Cancer Worry									
High	0.74	0.49–1.13	0.263	1.34	1.01–1.79	0.021	1.34	0.89–2.01	0.690
Moderate	1.12	0.77–1.62		1.58	1.20–2.08		1.17	0.78–1.76	
Low	1.00			1.00			1.00		

^a^ Comparison to the pre-contemplation group

Between the pre-contemplation and contemplation stages, only private cancer insurance was significantly different; women who had private cancer insurance were more likely to be in the contemplation stage than the pre-contemplation stage. In comparison of the pre-contemplation and action/maintenance stages, women aged 40–59 years who had private cancer insurance and who reported higher levels of cancer worry were more likely to stay in the action/maintenance stage than the pre-contemplation stage. However, between the pre-contemplation and relapse risk/relapse stages, no statistically significant difference was noted.

## Discussion

In the present study, we assessed the associations between cancer worry and stages of adoption for breast cancer screening in the general Korean population. Although previous studies have investigated the importance of the role of breast cancer worry in promoting participation in cancer screening [7–10], few studies have examined the relationship between breast cancer worry and stages of adoption for cancer screening in Korea. As we discovered, Korean women are indeed concerned with getting cancer, as 26% of the women included in this study responded that they always or very frequently worry about getting cancer. Specifically, women aged 60–69 years and women knew of the existence of the NCSP reported significantly higher levels of cancer worry than their counterparts. These results agree with previous studies that have suggested that women at greater risk for breast cancer or those with a family history of any cancer shows higher level of cancer worry [9, 27]. As described in Table 2, women in this study were also significantly more worried about getting cancer than other non-communicable diseases, including Alzheimer’s, hypertension, stroke, cardiovascular disease, and diabetes.

Across the stages of adoption, several factors were shown to influence differences in the distribution thereof in the present study. Mainly, subjects who reported relatively greater concerns for getting cancer were more likely to be classified in on-schedule stages (contemplation and action/maintenance). Additionally, age, monthly household income, private cancer insurance, undergoing regular health checkups, and cancer worry were significantly different across the stages of adoption for breast cancer screening. Meanwhile, perceived risk showed no significant difference across the stages of adoption. Reviewing previous studies, the effect of perceived risk on intention to undergo mammography was still unclear, though it has been studied as a promoter of preventive health behavior [28–31]. For example, one study from Hong Kong reported that perceived susceptibility of breast cancer did not show any meaningful contribution to mammography uptake in a population-based study [29]; however, in one Iranian female health belief study, women with greater perceived risk were more likely to report an intention to undergo screening [30]. These differences might be associated with ethnic differences. Kim et al. reported significant ethnic differences in how risk for cancer is perceived in an ethnically diverse sample of women, and showed that Asian women consistently had the lowest perceived risk of cancer, to the point that half of the respondents specified no risk [31]. Also, marital status showed no significant difference across the stages of adoption, although it is known to influence screening behavior; this is probably due to our small sample size, however.

In summary of the percent distribution across the stages of breast cancer screening adoption for Korean women, only about 63.8% reported on-schedule screening (contemplation 13.0%, action/maintenance 50.8%), while 36.2% did not (pre-contemplation 24.7%, relapse risk 8.9%, andrelapse 2.6%). These screening rates are much lower than those for on-schedule screening in the United States (84.0%) [32]. One previous study measuring on-schedule mammography rescreening rates in Korea indicated that several factors are associated with on-schedule screening [33]. Women of lower socioeconomic status, who had experienced receiving a false positive result, or were over the age of 70 years were less likely to beon-schedule; in contrast, women with previous experience with mammography screening were more likely to be classified as beingon-schedule [33]. The current study also showed that women with lower monthly household income or those older than70 years were less likely to be in on-schedule stages.

Among our results from multinomial logistic analysis, we found that cancer worry played a role in boosting cancer screening behaviors overall. Compared to those with relatively low cancer worry, subjects with relatively high levels of cancer worry were more likely to be classified in the action/maintenance stage than the pre-contemplation stage. Between the pre-contemplation stage group and the relapse risk/relapse stage group, those who reported relatively greater concerns for getting cancer were more likely to be classified in the relapse risk/relapse stages, suggesting that women with little to no concerns for getting cancer had not even considered undergoing breast cancer screening, in the past or the future. As introduced above, despite the existence of several contradictory results, cancer worry has predominantly been shown to play a critical role in motivating screening behavior, with which our results agree [9, 34]. Among studies that have analyzed the stages of adoption of cancer screening behavior in relation to cancer worry, one Japanese study reported an insignificant relationship between cancer worry and mammography adoption; however, the authors still suggested that cancer worry may shape goal intentions, implementation intention, perceived risks, and attitudes, thereby boosting positive screening behavior [35]. These contracting results may stem from differences in defining worry of getting cancer [34]. When the Cognitive-Social Health Information Processing (C-SHIP) model was adopted to explain the contradictory roles of cancer worry, it proposed an inverted U-shaped relationship between cancer screening and cancer worry, suggestingscreening intentions increase first, achieve apeak, and then decrease as cancer worry goes up [9]. Therefore, efforts to outline the entire scope of changes in intentions according to different levels of worry are needed. Additionally,several demographic factors have been shown to be associated with cancer worry. According to aprevious study, individualswithout educational qualifications and those from ethinic minority backgrounds showed higher level of cancer worry [34]. Therefore, further reviews and studies of the influences of cancer worry on the stages of adoption for cancer screening are needed.

Herein, we also examined socio-economic factors in relation to their effects on breast cancer screening behavior. In doing so, we discovered that Korean women aged 40–59 years, who are at high risk for breast cancer, are more likely to remain in the action/maintenance stages than the pre-contemplation stage (*P* for trend = 0.001). Additionally, women who had private cancer insurance and had higher household income (*P* for trend = 0.051) were more likely to be in the action/maintenance stage that the other stages, potentially indicating a health disparity related to the economic status of women.

Our study has a number of limitations. First, the cross-sectional design of this study hindered our ability to implicate any causal relationship for the observed associations. Thus, future studies with a longitudinal design should be conducted to track patterns in breast cancer screening behaviors. Second, because of memory bias, we assessed only the most recent breast cancer screening event and did not collect information on screening events that preceded the most recent one. Therefore, we could not distinguish between the action and maintenance stages. Further, we only measured overall cancer worry not breast cancer-specific worry, as we were concerned that there might be somewhat different trends between overall cancer worry and breast cancer-specific worry. Thus, studies that distinguish between worry for individual types of cancer are needed, as are validation studies to develop reliable and capable measures of predicting unique variances in cancer-relevant worry in the Korean population.

Despite these limitations, this study is important in that it is the first to assess breast cancer worry specifically in relation to its effects on screening behavior among Korean women. As well, this study provides new insights that may be of use in guiding the development of intervention strategies for improving compliance with breast cancer screening recommendations at the population level. Our results demonstrated that cancer worry is associated with continued breast cancer screening among women in Korea. Additionally, we showed that age, household income, private cancer insurance, and regular health checkups are positively associated with the stages of breast cancer screening adoption among Korean women. Interventions to address breast cancer worry may play an important role in increasing participation and equity in breast cancer screening.
